# Evaluation of data representation techniques for vibration based road surface condition classification

**DOI:** 10.1038/s41598-024-61757-1

**Published:** 2024-05-21

**Authors:** E. Raslan, Mohammed F․ Alrahmawy, Y. A. Mohammed, A. S․ Tolba

**Affiliations:** 1 New Damietta Institute for Engineering & Technology, New Damietta, Egypt; 2 Faculty of Computer and Information, Mansoura University, Mansoura, Egypt; 3 Faculty of Computer Science & Engineering, New Mansoura University, Gamasa, 35712 Egypt; 4 University of Economics and Human Sciences, Warsaw, Poland; 5 New Heliopolis Institute for Engineering & Automotive and Energy Technologies, New Heliopolis, Egypt

**Keywords:** Road surface condition classification, Time domain, Frequency domain, Time–frequency domain, Computer science, Civil engineering

## Abstract

The accurate classification of road surface conditions plays a vital role in ensuring road safety and effective maintenance. Vibration-based techniques have shown promise in this domain, leveraging the unique vibration signatures generated by vehicles to identify different road conditions. In this study, we focus on utilizing vehicle-mounted vibration sensors to collect road surface vibrations and comparing various data representation techniques for classifying road surface conditions into four classes: normal road surface, potholes, bad road surface, and speedbumps. Our experimental results reveal that the combination of multiple data representation techniques results in higher performance, with an average accuracy of 93.4%. This suggests that the integration of deep neural networks and signal processing techniques can produce a high-level representation better suited for challenging multivariate time series classification issues.

## Introduction

Road surface deterioration due to climate change, frequent use, heavy loads, and aging infrastructure can cause harm to pedestrians, drivers, and vehicles^1,2^. Moreover, monitoring these deteriorations using traditional approaches often requires considerable time, effort, and resources, delaying necessary repairs and potentially leading to accidents^2,3^. Therefore, efficient detection of road surface conditions is essential for ensuring the safety of drivers and pedestrians alike. Moreover, accurate detection of road surface conditions can also enable the development of smart transportation systems that can provide real-time updates to drivers, helping them make informed decisions while on the road, and to the authorities so they can take timely actions to prevent accidents and address potential risks.

Road surface quality detection can be achieved through three main techniques: (a) laser scanning, (b) computer vision, and (c) sensor-based^4^. Each of these techniques has its own strengths and weaknesses, making them appropriate for different situations and environments. Laser scanning: This technique offers high-precision 3D data of the road surface, which allows for the detection of small defects and unevenness. However, its effectiveness can be affected by weather conditions and requires specialized equipment, making it a less cost-effective option for widespread deployment. By using cameras installed on vehicles, computer vision utilizes road surface images to detect cracks, potholes, and other anomalies. This approach is relatively inexpensive and can be adopted during regular traffic flow. However, its accuracy can be affected by shadows, lighting conditions, and obscuring objects. Sensor-based: This approach utilizes sensors mounted on vehicles to capture vibration data caused by road surface irregularities. Sensor-based approaches are less susceptible to weather or lighting conditions and can be embedded into existing vehicle infrastructure. Additionally, sensor-based approaches can provide real-time data for immediate analysis and response, making them a powerful tool for proactive maintenance. However, they might have difficulty identifying different types of road defects and require feature extraction techniques for optimal performance.

Recently, with technological improvements in sensor technology and data processing capabilities, monitoring road conditions using sensor-based methods has become increasingly widespread due to their non-invasiveness and cost-effectiveness^2,5^. While time-domain analysis forms the foundation of many sensor-based approaches, it might not always accurately represent the full spectrum of information hidden within the complex vibration signatures generated by different road conditions. In order to address these shortcomings, this paper explores various data representation techniques that aim to extract richer features from the vibration data. These techniques go beyond the time domain and capture the information in the frequency domain, or the time–frequency domain, potentially leading to a more robust classification system.

The rest of the paper is organized as follows: in Sect. "Related work", an overview of the related work in vibration-based techniques for monitoring the condition of roads is provided. Section "Methodology" outlines the methodology employed in this study, including the acquisition and preprocessing as well as the architecture of machine learning models. Section "Results and discussion" presents, and discusses the experimental findings and performance assessments of diverse data representation techniques and machine learning models. Finally, in Sect. "Conclusion", we concludes this paper.

## Related work

Road surface quality significantly impacts vehicle safety, driving comfort, and maintenance costs. Vibration sensors mounted on vehicles offer a promising approach for non-destructive road condition assessment^3,6,7^. Several studies have explored the use of vibration sensors for road surface condition monitoring. Common sensor choices include accelerometers, gyroscopes, and magnetometers. The data collected from these sensors reflects the vehicle's response to road surface irregularities, such as potholes, cracks, and bumps^1–3,5,8^. The choice of sensors and their placement significantly impacts the data collected. Studies have explored using single-axis accelerometers for basic classification tasks and multi-axis configurations (accelerometer and gyroscope) for capturing richer information about road features^5,9,10^. The placement of vibration sensors on the vehicle can influence the sensitivity to specific road anomalies. Studies have investigated the effectiveness of sensors mounted on dashboards, floorboard, axles, wheels, and the vehicle chassis^10–12^. Vibration data often requires preprocessing steps like noise filtering and smoothing techniques to improve its quality and consistency before feeding it into machine learning models. These techniques help to remove irrelevant information and ensure features are on a similar scale for effective learning^1,11,13^. Due to the varying lengths of road sections with different surface conditions, raw vibration data is often segmented into windows for analysis. Techniques like GPS timestamps or signal characteristics are used for segmentation^4,11^.

Regarding classification approaches, machine learning algorithms play a vital role in classifying road surface types based on the extracted features. There are three main approaches to classifying road surface conditions based on vibration data: Rule-based methods, traditional machine learning-based methods, and deep learning-based methods^1^. Initially, early Rule-based approaches relied on various thresholds to detect road anomalies by setting predefined thresholds for signal characteristics like amplitude^1^. These thresholds are typically determined based on prior knowledge or empirical data^3^. Eriksson et al.^14^ used a threshold-based filter to identify potholes using acceleration and GPS data. They employed five thresholds (speed, high-pass, z-peak, x–z-ratio, and speed vs. z-ratio) to filter out the non-pothole data. Mednis et al.^9^ found that 3-axis acceleration data converges to zero when a vehicle passes over a pothole. They proposed a G-ZERO algorithm and compared it with three other heuristic threshold methods (Z-THRESH, Z-DIFF, and STDEW) for detecting potholes, achieving an accuracy rate of 90%. Astarita et al.^15^ focus on accelerometer data to identify the patterns associated with speed bumps and potholes. By examining the extreme peaks of the z-axis, they were able to detect speed bumps with 90% accuracy and potholes with 65% accuracy. In conclusion, while the threshold-based approach is easy to implement, it requires determining reliable thresholds through numerous experiments. Additionally, due to variations and the need for frequent adjustments, it becomes challenging to apply this approach to large-scale road surface detection.

Traditional machine learning algorithms have been utilized to enhance the accuracy and reliability of road surface condition detection^11^. These algorithms are capable of learning and adapting to different scenarios through training on various road types and conditions^1,3^. To detect speed bumps, Celaya et al.^5^ suggested extracting statistical features like the mean, variance, and standard deviation from X-axis and Y-axis gyroscope and Y-axis accelerometer data. They employed logistic regression and showed an accuracy of 97.14%. In another study, Ferjani et al.^16^ tested support vector machines, decision trees, and multilayer perceptrons to investigate the characteristics of the time and frequency domains for road monitoring using three-axis accelerometer data. They trained a decision tree model to detect potholes, metal bumps, asphalt bumps, and worn-out roads with an accuracy of 94.00% using both time-domain features (mean, variance, standard deviation, median, entropy, and more) and frequency-domain features (spectrum energy, median frequency, minimum magnitude, and more). Wu et al.^17^ proposed extracting features from the three axes of the accelerometer data in time domain, frequency domain, and time–frequency domain representations. They employed a random forest classifier to identify road potholes, achieving an accuracy of 95.7%, a precision of 88.5%, and a recall of 75.0%. Additionally, the study conducted by Zhou et al.^18^ focused on classifying the quality of manholes based on time and frequency domain features extracted from accelerometer and gyroscope data. They used a support vector machine to categorize manholes into three classes: good, average, and poor, which correspond to different levels of subsidence. The study reported an average classification accuracy of 84.40%.

Deep learning techniques, especially recurrent neural networks (RNNs) and convolutional neural networks (CNNs), have demonstrated effectiveness in dealing with multivariate time series classification problems^19^. RNNs are designed to capture temporal dependencies in sequential data, while CNNs are effective at capturing spatial features from sensor data. These networks have the capability to be trained end-to-end, which allows them to learn feature representations directly from the raw sensor signals without applying any signal transformation^1,3,19^. Varona et al.^20^ compare reservoir computing models, CNNs, and LSTMs by processing the smartphone's accelerometer data to automatically identify potholes and destabilizations caused by speed bumps or driver actions. With 85% accuracy, the CNN model easily surpassed the other deep learning techniques. Further, Tiwari et al.^21^ proposed a CNN-based method for the classification of road surface quality (good road, medium road, and bad road) using accelerometer data as input. The proposed method exceeded neural feedforward networks and support vector machines (SVM), achieving a precision of 98.5%. To classify roads into the categories of good, fair, and poor, Sabapathy et al.^22^ evaluated the ordinal logistic model, the SVM model, the ANN model, and the CNN model using accelerometer and speed data collected from OBD-II. The CNN model's overall accuracy on the validation dataset was 65.6%, but it outperformed others. In conclusion, the effectiveness of deep neural networks in addressing complex multivariate time-series classification challenges is evident. However, there is potential for further improvement by exploring novel methods that combine deep neural networks with advanced signal processing techniques to produce higher-level representations for these challenging tasks problems.

## Methodology

We collect a multivariate time series dataset of vibration data from sensors mounted on vehicles. Multivariate Time Series Classification (MTSC) is a significant challenge in machine learning and has many practical applications across various domains. In order to train machine learning models to classify road surface conditions, there are a number of representations that can be used to represent the time series signals^6^. We focus on the following data representation domains: the time domain, the frequency domain, and the time–frequency domain. Finally, deep learning algorithms are employed to evaluate the effectiveness of various data representations and the models' ability to accurately classify complex patterns.

This section is organized as follows: In Sect. "Dataset collection", we discuss the process of collecting the dataset for our study. Section "Data representation transformation" focuses on the transformation of the collected data into a suitable representation format for deep learning models. Moving on to Sect. "Deep learning models", we present the various deep learning models employed in our study and their architectures. In Sect. "Experiment setup", we outline the experimental setup, including details about how we handled the imbalanced dataset.

### Dataset collection

**Figure 1:**
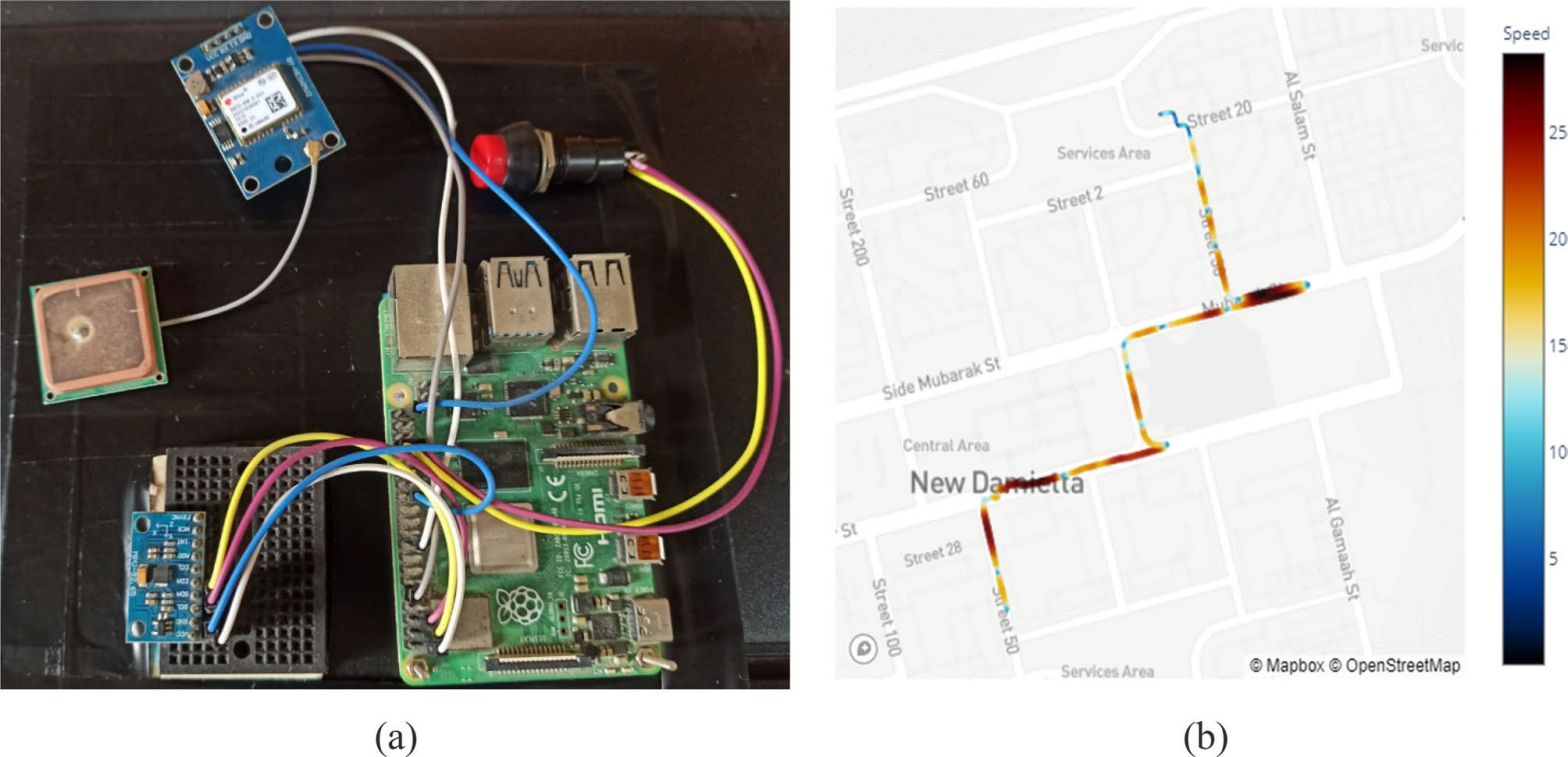
(**a**) Data acquisition system mounted on the vehicle dashboard, (**b**) Illustrative map depicting a sample trip used for data collection.

A Raspberry Pi 4 served as the central processing unit for the data acquisition system. This computer was connected to an MPU 9250 9-DoF IMU (Inertial Measurement Unit) sensor and a Neo 6m GPS unit, as shown in Fig. 1a. The entire system was fixed to the dashboard of the vehicle for data collection. The vehicle-mounted accelerometer used in this study collected data at a sampling frequency of 1kHz. The data collection process involved driving the instrumented vehicle on various road segments categorized into four main classes: normal road surface, potholes, bad road surface, and speed bumps. Data was collected at different speeds form a diverse set of Egyptian asphalt roads at different speeds and in real-world scenarios to account for the influence of vehicle velocity on vibration patterns, as shown in Fig. 1b.

**Figure 2:**
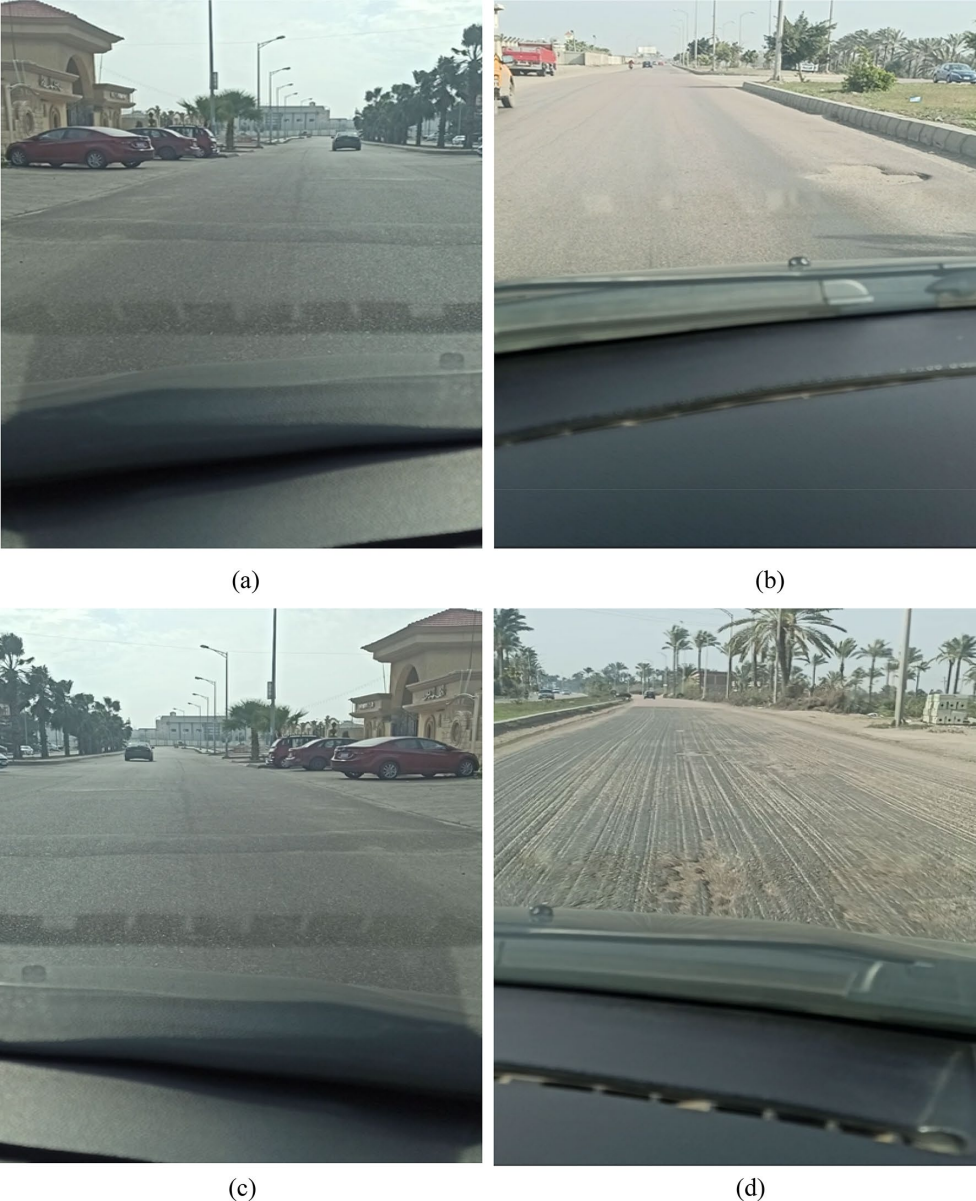
Representative data samples for different road conditions. (**a**) Normal road, (**b**) Pothole, (**c**) Speedbump, (**d**) Bad road.

Figure 2 showcases various road conditions. The normal road exhibits a smoother, less fluctuating pattern compared to the pothole, which shows a sharp drop in acceleration followed by a rapid increase. Similarly, the speed bump displays a characteristic hump-shaped pattern reflecting the vehicle's ascent and descent. Compared to a smooth road with a consistent pattern, the accelerometer readings from a bad road will exhibit more frequent and irregular variations. These fluctuations reflect the vehicle's response to the constant changes in elevation.

Sensors' raw data is frequently represented as a time series, where the values of the data are represented as points that are recorded at regular intervals. Six time series are recorded for roads with four different conditions (normal, bad, speed bump, and pothole): three for X, Y, and Z-axis acceleration values,and three for pitch, roll, and yaw. The accelerometer data reflects the vibration patterns experienced by the vehicle due to the road surface irregularities. while the gyroscope readings can potentially capture subtle changes in vehicle orientation related to the road conditions.

**Figure 3:**
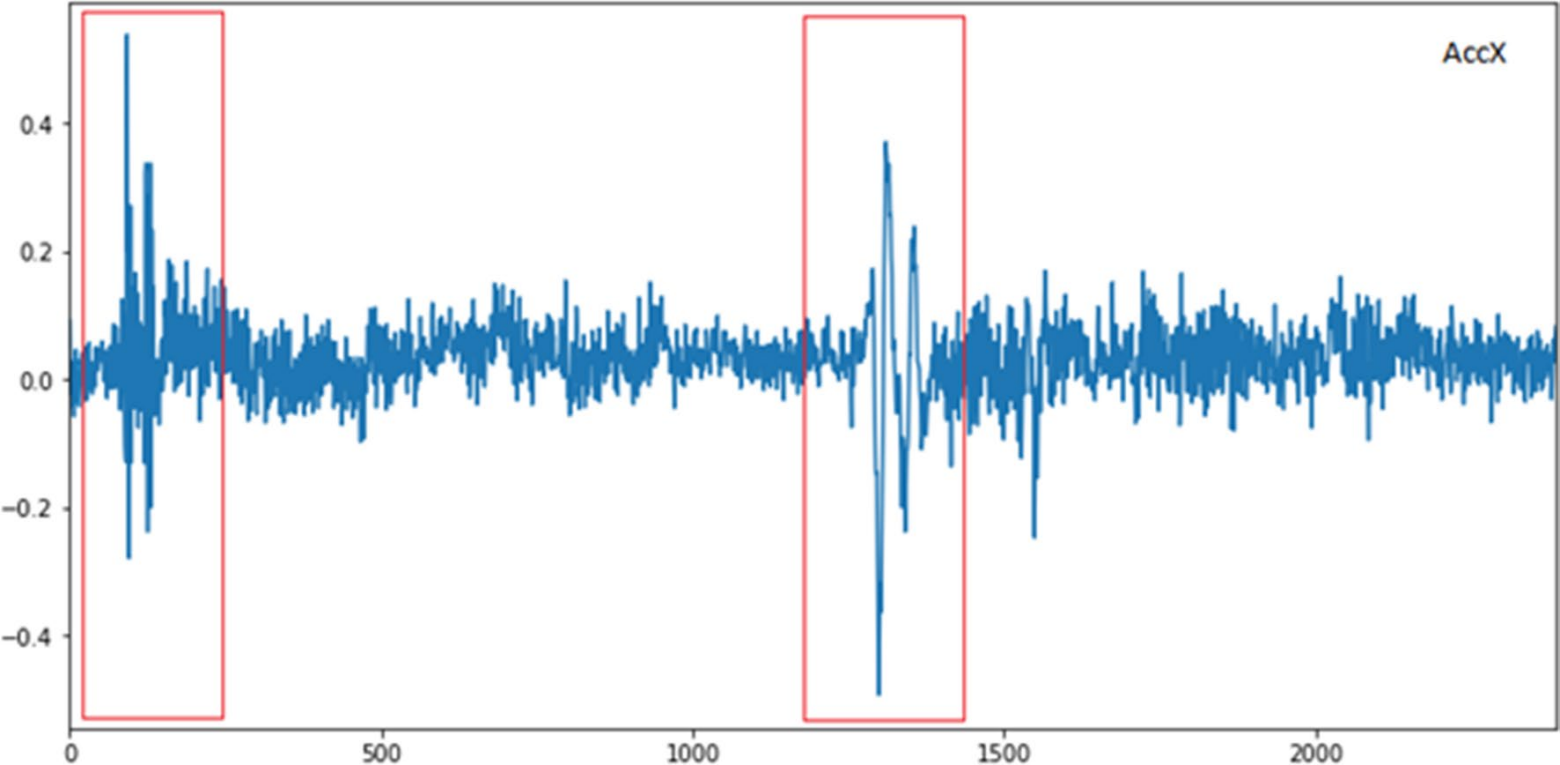
X-axis acceleration signatures for potholes and speed bumps.

By analyzing the time series data, it is possible to identify patterns that can provide insights into road conditions. Figure 3 highlights the distinct vibration patterns associated with Pothole and Speed Bump. First Red Segment exhibits a distinct signature characterized by a sharp drop in X-acceleration followed by a rapid increase. This pattern corresponds to the vehicle encountering the sudden depression of the pothole and its subsequent recovery. while the second red segment shows a characteristic hump-shaped pattern. The initial rise represents the vehicle ascending the speed bump, followed by a dip as it traverses the peak, and finally a rise as it descends the other side.

**Figure 4:**
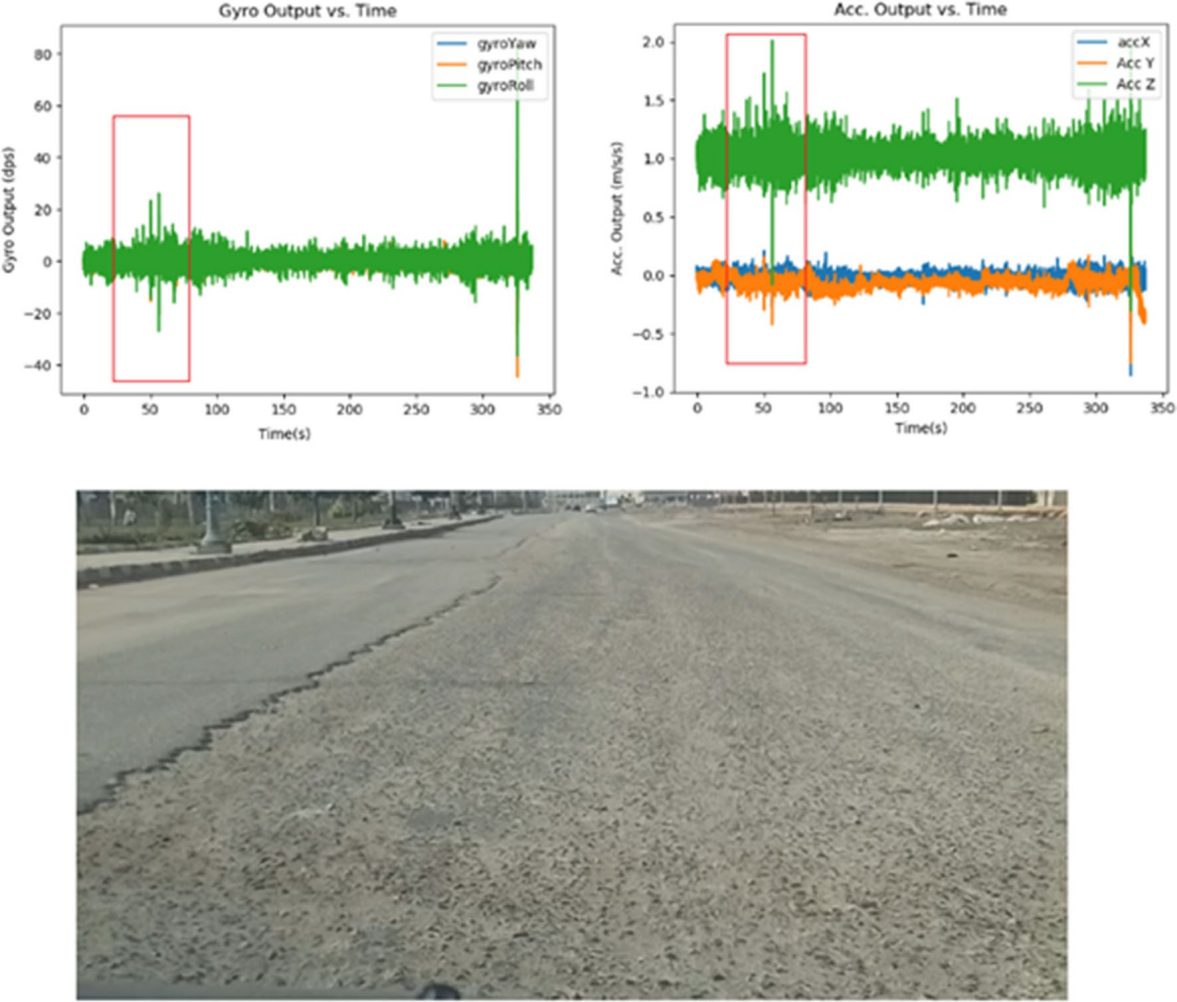
Time series data sample for a bad road segment.

On the other hand, Normal Road segments display a relatively smooth and consistent pattern with minimal fluctuations. while, The bad road data showcases a more erratic pattern compared to the other categories. It might involve frequent fluctuations, higher peak accelerations, and potential sharp spikes or dips due to the uneven surfaces characteristic of bad roads as shown in Fig. 4.

**Figure 5:**
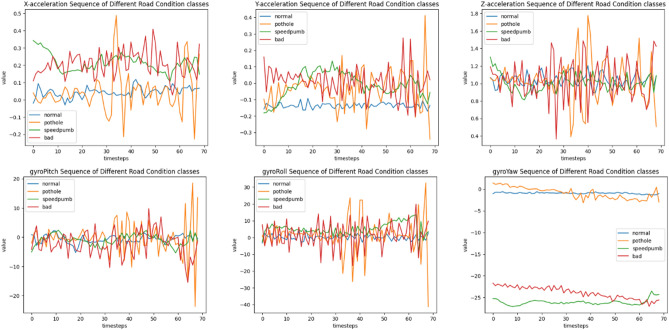
Resampled time-domain data samples for different road conditions.

To prepare the data for classification, Time Series signals are combined into a single multivariate dataset, where each row represents a time step and each column corresponds to a signal value. Furthermore, we need to divide the road into segments so that each segment can be classified individually. So, location-based windows of sensor data streams were formed. Since GPS data is only collected once every second, while IMU data is typically collected once every 14 ms, the GPS data collection points act as boundaries around the IMU data. The maximum number of data points in each window, represented by the GPS coordinates, was 74. So, After eliminating all time series with fewer than 50 samples, each sequence was resampled to fit the 74 timesteps by interpolating the data from the closest data points, As shown in Fig. 5. The result is that the roads are segmented, and each segment of a road can be classified separately.

The dataset was created through manual labeling of captured videos from various experiments. Prior to manual labeling , we conducted extensive exploratory data analysis to gain insights into the characteristics of the vibration data. This analysis involved employing various data visualization techniques. These exploratory analyses provided valuable insights and laid the groundwork for our subsequent classification efforts. Additionally, we experimented with clustering algorithms such as k-means and DBSCAN to automatically group similar data points. Nevertheless, additional research revealed that these clustering techniques were unable to accurately differentiate between the wide variety of road surface conditions included in our dataset. To supplement our analysis and aid in precise annotation, we integrated multiple data sources, including video recordings of road segments. These video recordings offered contextual information and were utilized as an additional reference during the annotation process. Our classification process followed an iterative approach, wherein we continuously refined our annotation methodology based on insights gained from the data, which ensured the accuracy and consistency of our annotations across different road surface conditions.

### Data representation transformation

Extracting valuable features from the sensor data is essential for training machine learning algorithms to accurately classify road surface conditions. Three main data representations are used to depict these multivariate time series signals: time domain, frequency domain, and time–frequency domain^6^.

Road surface conditions are represented by the raw vibration sensor data that has been gathered over time. However, this representation requires significant feature extraction to capture hidden information, as the raw signal might contain noise and irrelevant fluctuations^8^. Frequency domain analysis, using techniques like the Fast Fourier Transform (FFT), helps figure out the distribution of energy across different frequencies within the vibration signal^23,24^. By decomposing the signal into its component frequencies, FFT allows us to identify the most prevalent frequencies present and potentially associate them with specific road surface characteristics. For example, vibrations caused by potholes may occur at specific frequencies that are different from those produced by smoother segments of the road^23^. Time–frequency domain analysis techniques like discrete wavelet transform (DWT) and continuous wavelet transform (CWT) offer a more comprehensive representation compared to pure time or frequency domain analysis. These techniques decompose the signal into wavelets, allowing analysis of both the frequency component and its variation over time. This capability can be beneficial for capturing transient features related to road anomalies, such as sudden bumps or potholes, which might not be explicitly detected in the time or frequency domain alone^23^.

**Figure 6:**
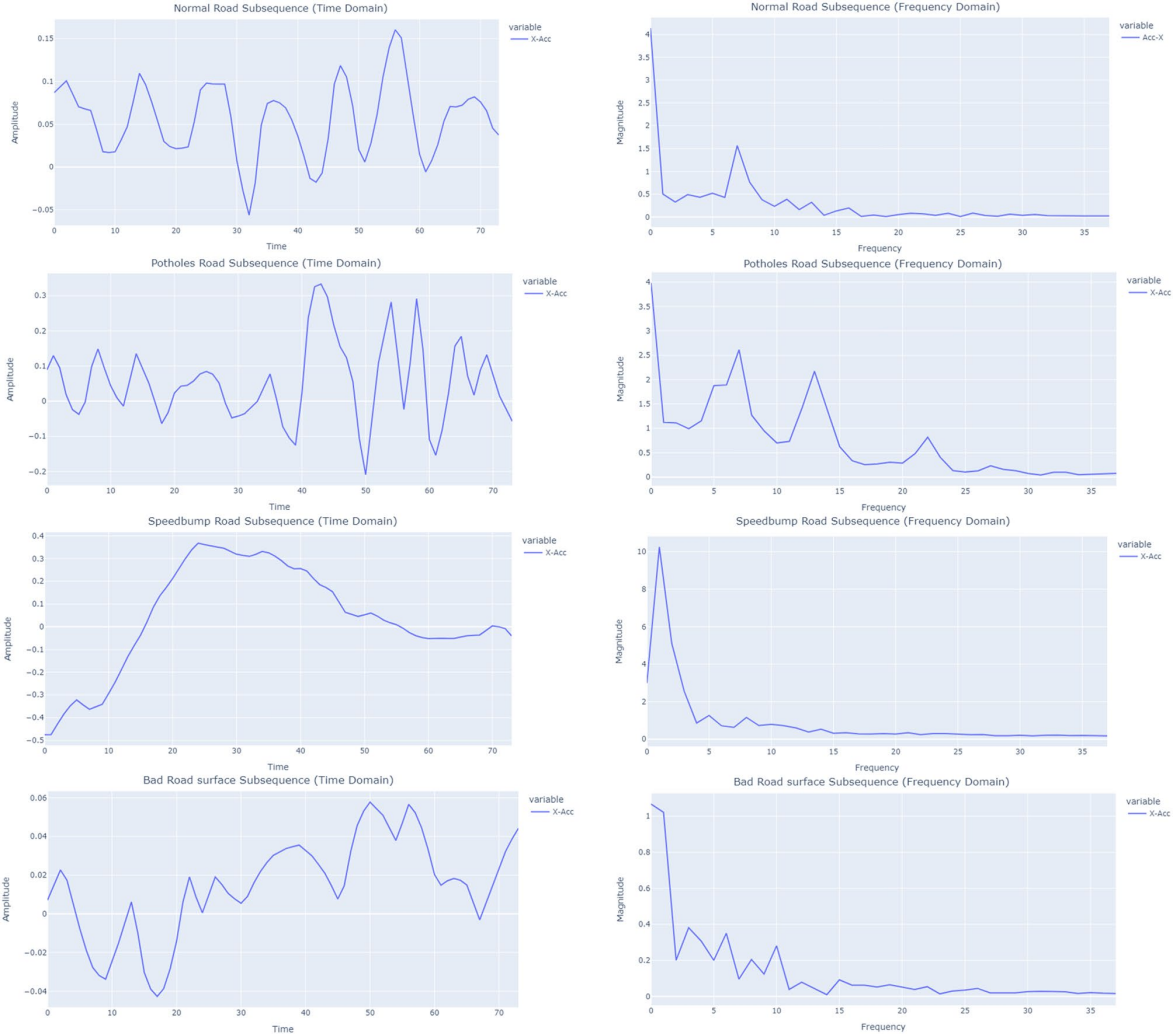
Comparison of various road surface conditions in the frequency and time domains.

In our experiment, we applied FFT to the sequences extracted from the time-series data. By taking the absolute value of the Fourier transform for each sequence, the magnitude of the frequency components present in the signal was determined. This allows us to identify the dominant frequencies and their magnitudes in the signal, as shown in Fig. 6. The shape of the result dataset (2105, 38, 6) showed that it consists of 2105 sequences, each with 38 time steps and 6 features.

**Figure 7:**
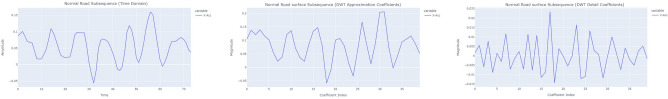
Normal road surface subsequence in the time and time–frequency domain using DWT.

The discrete wavelet transform (DWT) is a mathematical transformation used to break down a signal into a set of wavelets, which are small waves that can be utilized to represent the original signal^25^. The DWT works by passing the signal through a number of high-pass and low-pass filters, which produce a multi-resolution representation of the signal. We applied a single level DWT using the Daubechies 4 wavelet for each subsequence to transform our dataset from time domain to time–frequency domain. It separates the approximation coefficients (CA) and detail coefficients (CD). The shape of the CA or CD (2105, 40, 6) indicates that it consists of 2105 subsequences, each with 40 timesteps and 6 Features. In Fig. 7, the transformed coefficients, obtained through the DWT, are visualized. As indicated, CA represents a coarse representation of the original signal. On the other hand, CDs reveal fine details and sharp changes in the signal.

**Figure 8:**
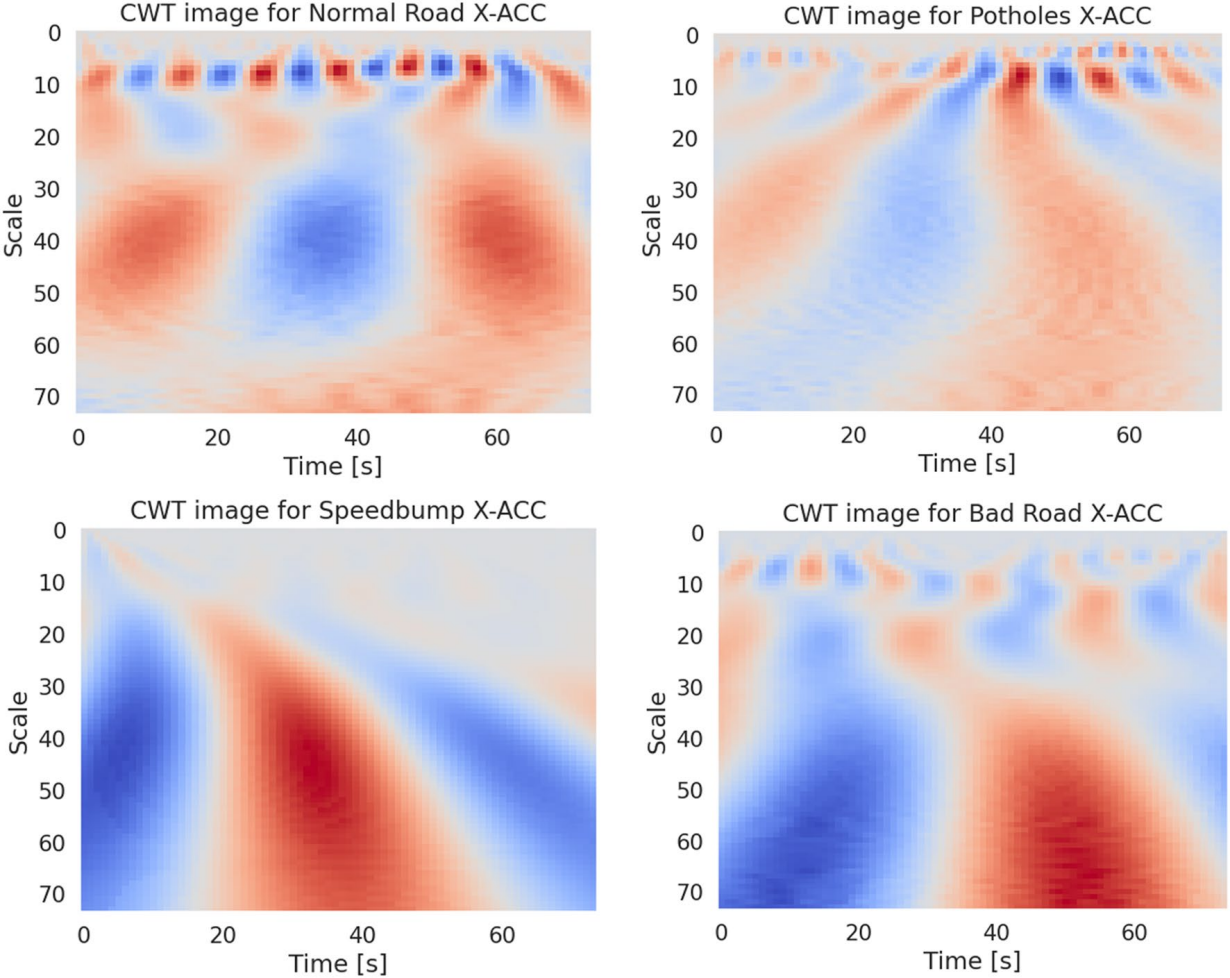
Road surface subsequences in the time–frequency domain using CWT.

The continuous wavelet transform (CWT) allows for the decomposition of a signal into its constituent frequency components, revealing both the frequency content and the evolution of the frequency content over time^24^. We applied CWT to each time series sequence separately, which involves mapping the frequency spectrum of each time series onto a 2D image. We employed Morlet wavelet With a scale of 74. Figure 8 demonstrates the image representation of the diverse road surface conditions.

### Deep learning models

To compare the effectiveness of the various data representations, we used deep learning algorithms, particularly long short-term memory (LSTM) and convolutional neural networks (CNN). These algorithms are widely applied in several fields, such as natural language processing, computer vision, and speech recognition. LSTM is especially effective in capturing long-term dependencies in sequential data, while CNN is particularly good at extracting spatial features from images. The subsections present the different models utilized in our study and their architectures.

#### LSTM (long short-term memory)

The LSTM is a recurrent neural network (RNN) architecture developed to capture long-term dependencies in sequential data^26,27^. A major benefit of LSTM models is their ability to avoid the vanishing gradient problem frequently encountered with traditional RNNs, which can prevent the learning process for long sequences^26,27^. By using a combination of input, forget, and output gates, LSTM units are able to manage the flow of information across the network, which allows for more effective learning of temporal patterns. As road surface conditions demonstrate dynamic and time-varying characteristics, LSTM networks are appropriate for modeling the temporal correlations present in the vibration data.

The model architecture is composed of two main layers. The first layer is an LSTM layer with 128 units. The input shape of this layer is a sequence of vectors with a fixed length. Each vector represents a sample in the input sequence. The second layer is a dense layer, which acts as the final stage of the model. The activation function used in this layer is softmax, which is typically used for multi-class classification problems.

#### 1D CNN

**Figure 9:**
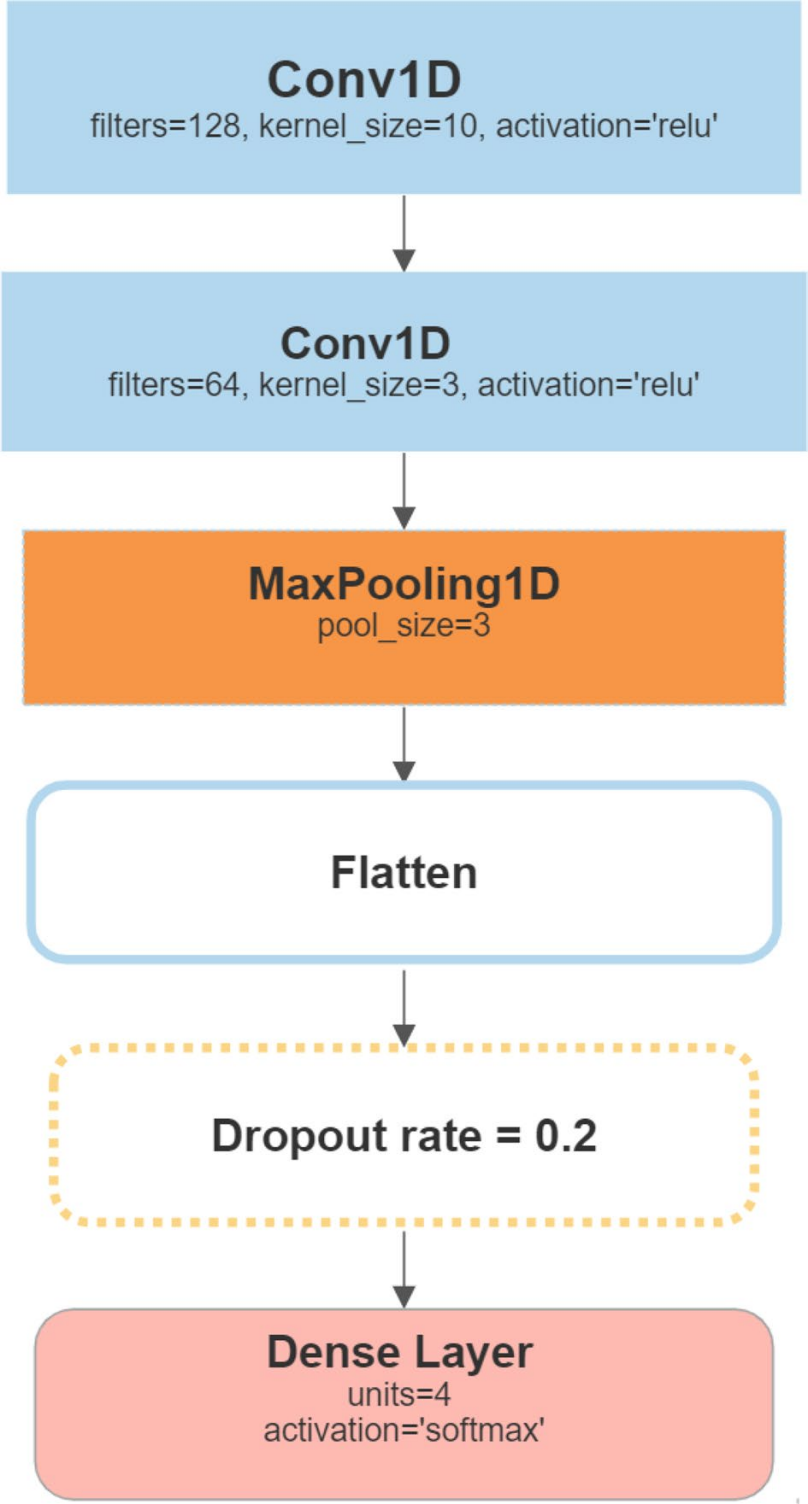
1D CNN model architecture.

The CNN model, originally designed for image processing, can also be adapted for multivariate time series classification by treating each time step as a channel^27^. The CNN model begins with a Conv1D layer with 128 filters and a kernel size of 10, which applies convolutional filters to the input sequence. The rectified linear unit (ReLU) activation function is used to introduce non-linearity. A second Conv1D layer with 64 filters and a kernel size of 3 follows the initial layer, further capturing local dependencies within the sequence. To reduce the dimensionality and extract the most relevant features, a MaxPooling1D layer with a pool size of 3 is employed. This layer downsamples the input representation by selecting the maximum value within a sliding window. Subsequently, a Flatten layer is applied. Additionally, Dropout regularization with a dropout rate of 0.2 is incorporated to prevent overfitting, and a final Dense layer with a softmax activation function is utilized for multi-class classification, see Fig. 9.

#### 2D CNN

There are several representations that can be used to represent a multivariate time series signal for feeding into a machine learning algorithm, including raw time series representation, feature-based representation, and image-based representation^28^. Multivariate time series data can be represented using an image-based representation by transforming the data into 2D images. Once the time series data has been mapped to an image, the data can be classified using common image processing methods like 2D CNN^28^.

**Figure 10:**
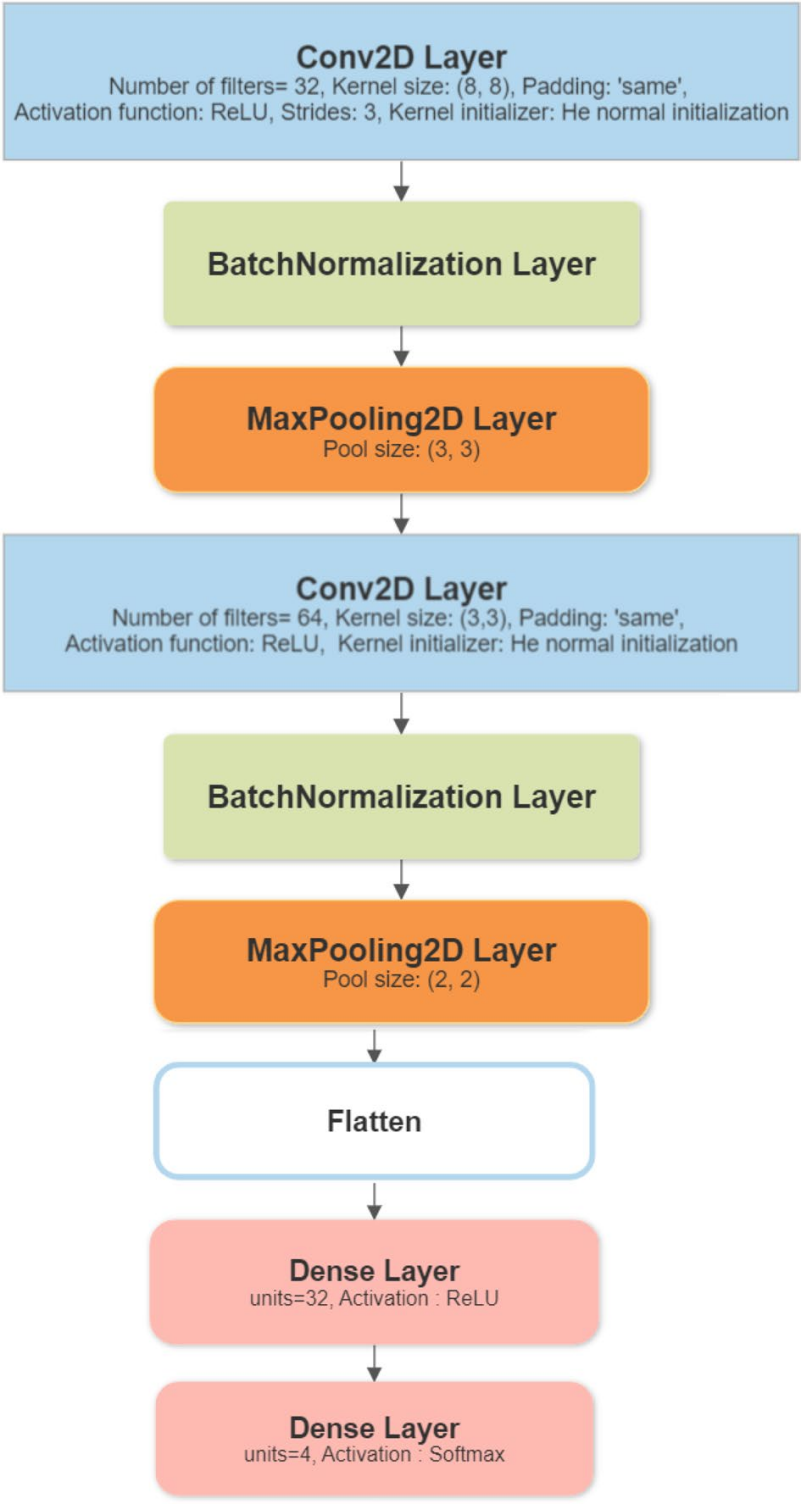
2D CNN Image-based model architecture.

As shown in Fig. 10, the 2D CNN image-based model architecture is as follows: The first Convolutional Layer comprises 32 filters with an 8-kernel size and employs a ReLU activation function. The layer applies 3-pixel strides and adopts the 'same' padding type. The kernel weights are initialized using the he-normal initialization method. To improve the stability and convergence of the network, a batch normalization layer is added after the first convolutional layer. After that, a Max Pooling Layer performs downsampling using a 3 × 3 pooling window to reduce spatial dimensions while preserving essential features. Followed by the second Convolutional layer, which consists of 64 filters with a kernel size of 3 × 3 and applies the ReLU activation function. No padding is used, and the weights are initialized using the he-normal initialization. Another batch normalization layer Similar to the previous one, it is added after the second convolutional layer to enhance network performance. Then Max Pooling performs another downsampling operation using a 2 × 2 pooling window. The output of the last pooling layer is flattened to create a vector representation of the extracted features. Finally, two fully connected layers are added to the network. The first fully connected layer consists of 32 neurons, which allows for a more complex mapping of the features. On the other hand, the second fully connected layer is made up of four neurons that represent the classes of road surface conditions.

#### Proposed model

**Figure 11:**
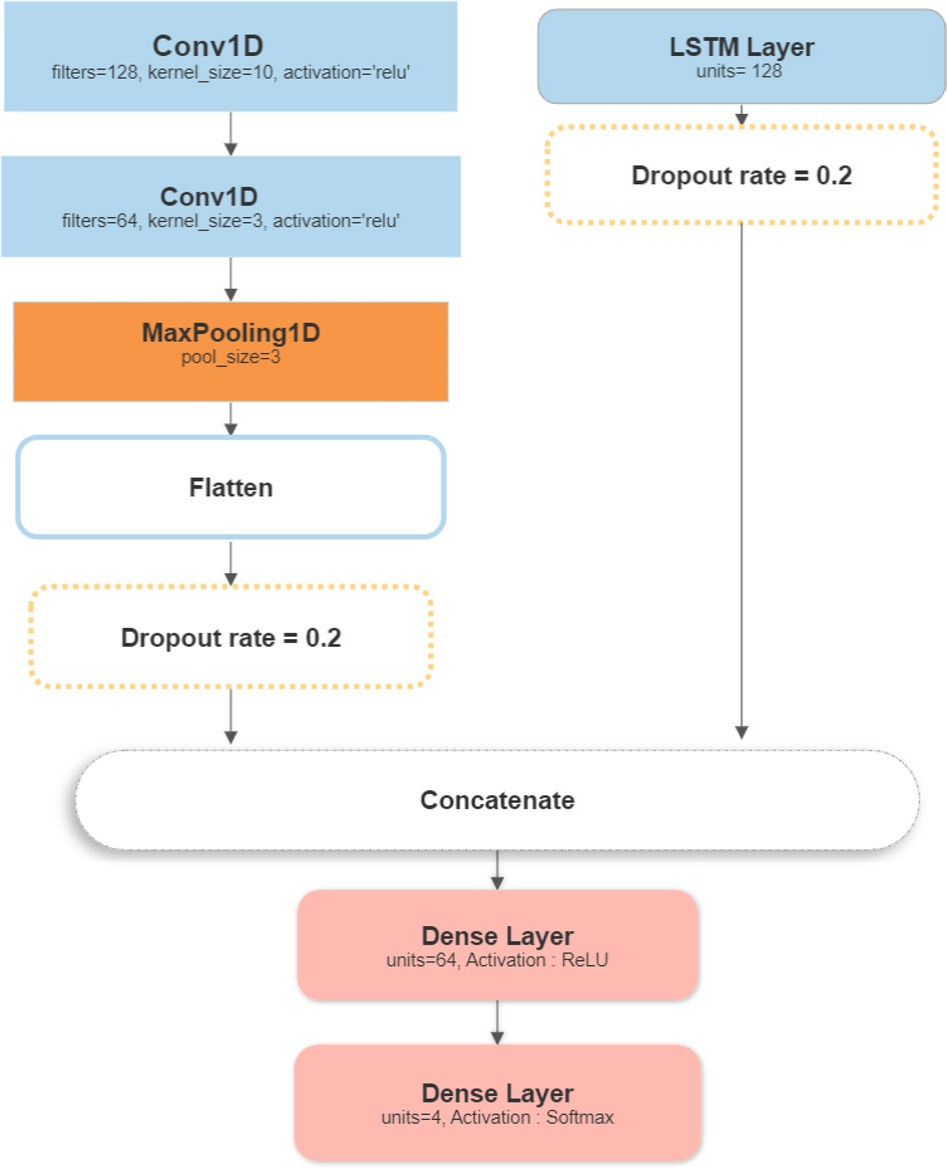
Proposed model architecture.

The proposed model consists of two branches: an LSTM branch and a CNN branch. The LSTM branch follows the architecture described in Sect. “LSTM (long short-term memory)”, while the CNN branch follows the structure described in Sect. “1D CNN”. The outputs from both branches are concatenated and passed through Two fully connected Dense layers. The first Dense layer has 64 units and uses the ReLU activation function, while The second Dense layer has 4 units (representing the number of classes) and uses a softmax activation function for classification (see Fig. 11).

### Experiment setup

Table 1 shows that the majority of the training dataset samples (86% of the training dataset samples) belong to the normal road surface class, while only a small proportion (0.7%, 2.7%, and 10.5%, respectively) belong to the potholes, speed bumps, and bad road surface classes. While this dataset is representative of real-world scenarios, it was biased toward normal road surface class.

**Table 1:** Imbalanced training and test dataset subsequences.

Class	Train dataset	Test dataset
Normal road surface	1048	378
Potholes	2	3
Speedbump	69	12
Bad Road surface	56	46
Total	1175	439

**Figure 12:**
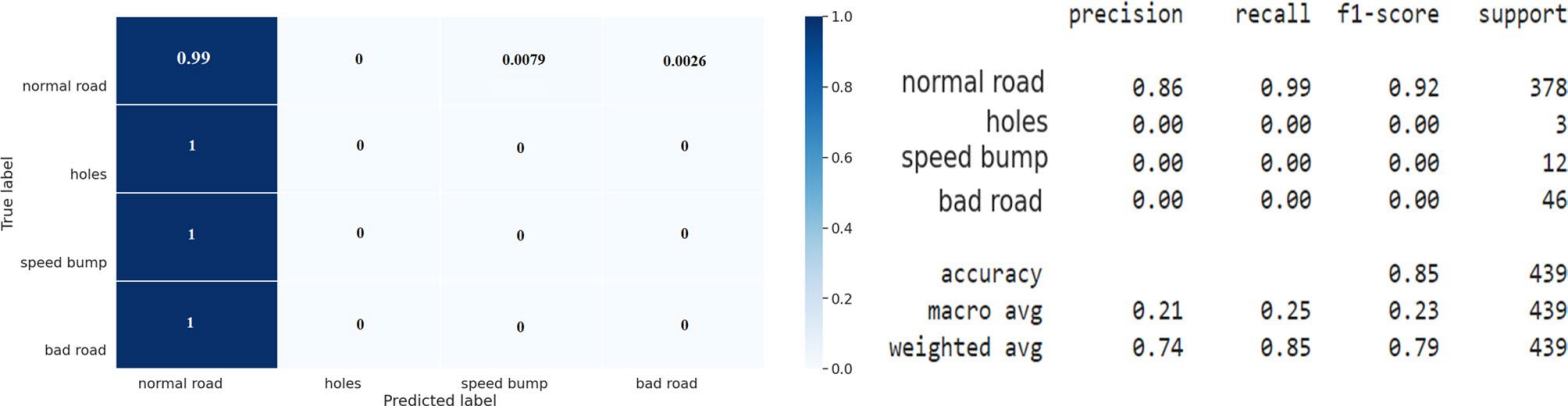
Confusion matrix and classification report of the imbalanced dataset.

The below figure (Fig. 12) shows the classification report and the confusion matrix of the performance of the 128-unit LSTM, followed by a dense layer to classify the various types of road surfaces. However, the LSTM model achieves 85% classification accuracy, The results indicate that the model struggles to differentiate between normal road surfaces and other classes; all potholes, speedbumps, and bad road surface segments were classified as normal road surface classes.

To address this issue, oversampling and undersampling techniques have been used to balance the dataset and improve the model's performance. The oversampling technique involves increasing the number of instances in the minority classes (i.e. potholes, speedbumps, and bad road surface), while the undersampling technique involves reducing the number of instances in the normal road surface class.

To keep track of the sample class, we divided all continuous samples that belonged to the same class into distinct groups. Then, using the TimeSeriesResampler, we doubled the size of each group in the dataset and ensured that the output would be a factor of sequence length (i.e. 74). Additionally, we performed the rolling window technique to increase the number of instances of the minority class, which involves sliding a window over the time series data and extracting subsequences of a fixed length from each position of the window, then labeling each subsequence with the same label as the original time series. We used different overlap fractions for each minor class to balance the dataset. Table 2 represents the training and testing subsequences for the modified dataset.

**Table 2:** Edited dataset subsequences.

Class	Number of subsequences
Normal road surface	831
Potholes	149
Speedbump	492
Bad Road surface	633
Total	2105

### Ethical and informed consent for data used

Not applicable [Given the nature of the data utilized, there were no human participants or volunteers involved in the data collection process. Therefore, no informed consent was required from individuals for the use of their data in this study].

## Results and discussion

This section investigates the effectiveness of data representation on road surface classification performance using LSTM and CNN models. We examine the models in three domains: time, frequency, and time–frequency. To examine the performance of our models, we used stratified k-cross validation sampling during the train-test split to guarantee a representative distribution of classes in both sets with k = 5. We trained our models using a sparse categorical cross-entropy loss function for 20 epochs with a batch size of 32 and compiled them with the Adam optimizer. Additionally, we employed performance metrics that are robust to imbalanced datasets, such as accuracy, precision, recall, and F1-score. Accuracy refers to classification accuracy, which measures the percentage of correctly classified instances out of the total number of instances. Precision measures the ratio of correctly predicted positive observations to the total predicted positives; recall measures the ratio of correctly predicted positive observations to all observations in the actual class; and F1-score is the harmonic mean of precision and recall. The experiments were performed on a dataset consisting of 2105 samples, where each sample represented a multivariate time series consisting of 6 series with 74 timesteps in the time domain.

### Time domain results

The LSTM model, which consists of an LSTM layer with 128 units and a dropout rate of 0.2 to reduce overfitting, achieved an average accuracy of 83.43% across the 5 folds with a standard deviation of 0.0305, an average precision of 83.9%, an average recall of 84.97%, and an F1-score of 84%. As presented in Table 3, normal road surface class and pothole class showed average F1-scores of 89.02% and 90.68%, respectively, indicating that the model was particularly effective at identifying these types of road conditions. However, the model struggled with speed bumps and bad roads, achieving F1-scores of only 79.4% and 76.9%, respectively.

**Table 3:** Comparing five-folds cross-validation average results for LSTM and CNN models using time domain representation.

Class	LSTM			1D CNN		
	Avg. precision	Avg. recall	Avg. F1-score	Avg. precision	Avg. recall	Avg. F1-score
Normal road	86.67	91.7	89.02	89.2	90.62	89.87
Potholes	86.96	95.32	90.68	90.23	93.34	91.27
Speedbumps	77.77	81.93	79.4	85.03	84.36	84.58
Bad road	84.2	70.94	76.9	81.36	79.15	80.21
macro avg	83.9	84.97	84	86.46	86.87	86.48
Avg. accuracy	83.43%			85.9%		
Std	0.0305			0.0157		

We also experimented with a CNN model that consisted of two 1-D convolutional layers, Max-Pooling layer and a dense layer. This model was trained using the same dataset and optimization technique as the LSTM model. The CNN model showed better results, with an average accuracy of 85.9% and a standard error of 0.0157. The CNN model achieved Average precision, recall, and F1-score values above 89% for the normal road surface class, although the potholes and speed bumps class experienced higher performance than the LSTM model (see Table 3).

### Frequency domain results

From the experimental results presented in Table 4, the CNN model is superior to the LSTM model in both the time and frequency domains and is able to extract more meaningful features from the data in both domains. When the dataset's frequency representation was evaluated, the CNN model had an average accuracy of 90.7%, which was better than the LSTM model's average accuracy in the frequency domain by 6% and the LSTM model's average accuracy in the time domain by 7.3%. Additionally, it performed better than the same model when using the time representation by 4.8%.

**Table 4:** Comparing five-folds cross-validation average results for LSTM and CNN models using frequency domain representation.

Class	LSTM			1D CNN		
	Avg. precision	Avg. recall	Avg. F1-score	Avg. precision	Avg. recall	Avg. F1-score
Normal road	92.73	90.98	91.8	91.67	94.71	93.13
Potholes	81.45	82.56	81.11	93.68	94.65	94.11
Speedbumps	76.09	89.03	81.91	87.11	92.7	89.74
Bad road	83.44	73.32	77.93	92.28	83.1	87.38
Macro avg	83.43	83.97	83.19	91.19	91.29	91.09
Avg. accuracy	84.6%			90.7%		
Std	0.0144			0.0060		

### Time–frequency domain results

The results presented in Table 5 illustrate the comparison of average performance metrics for LSTM and 1D CNN models using the discrete wavelet transform (DWT) with the Approximation Coefficients and Detail Coefficients separated. LSTM and 1D CNN models using DWT Approximation Coefficients are effective in classifying different road surface conditions. While both models show comparable performance, the LSTM model tends to achieve slightly higher precision, recall, and F1-scores across the classes, resulting in a slightly higher average accuracy compared to the 1D CNN model. The average Accuracy for all classes is 86.18% for the LSTM model and 85.32% for the 1D CNN model. Additionally, both the LSTM and 1D CNN models perform well in detecting potholes, with the highest F1-score, precision, and recall achieved for the Potholes class.

**Table 5:** Comparing five-folds cross-validation average results for LSTM and CNN models using DWT.

Class	Approximation coefficients						Detail coefficients					
	LSTM			1D CNN			LSTM			1D CNN		
	Avg. precision	Avg. recall	Avg. F1-score	Avg. precision	Avg. recall	Avg. F1-score	Avg. precision	Avg. recall	Avg. F1-score	Avg. Precision	Avg. recall	Avg. F1-score
Normal road	90.13	91.58	90.8	88.75	91.1	89.73	84.51	90.7	87.3	81.33	86.77	83.93
Potholes	91.82	95.66	93.41	93.48	92.63	92.99	82.96	79.19	80.22	86.04	74.58	79.67
Speedbumps	83.28	85.78	84.35	81.03	85.37	82.92	76.58	84.55	80.01	67.61	77.84	72.32
Bad Road	84.29	79.06	81.49	82.65	76	79.14	86.14	68.92	75.21	76.17	62.39	68.42
Macro avg	87.38	88.02	87.51	86.48	86.27	86.2	82.55	80.84	80.68	77.79	75.4	76.08
Avg. Accuracy	86.18%			85.32%			72.2%			76.5%		
Std	0.0190			0.0143			0.0209			0.0289		

Comparing these results with the results obtained using high-frequency data (DC), we find that there are significant differences in the performance. The low-frequency data provides a much more detailed picture of the underlying trends and patterns, allowing for more accurate classification. For the LSTM model, The average accuracy of the LSTM model was 72.2%, with a standard deviation of 0.0209. The average accuracy of the 1D CNN model was 76.5%, with a standard deviation of 0.0289.

The results presented in Table 6 compare the average performance of LSTM and 1D CNN models using both DWT coefficients with different approaches. The first approach is to use CA and CD concatenated; the LSTM model achieved an average accuracy of 81.43% for all the classes, while the 1D CNN model achieved a higher accuracy of 86.4%. Similarly, the 1D CNN consistently outperformed the LSTM model in terms of macro-average precision, recall, and F1-score. When examining the results for the CA and CD stacked, the LSTM model demonstrated a higher performance compared to the concatenation approach and achieved a higher average accuracy compared to the 1D CNN model. The average accuracy of the LSTM model with the CA and CD stacked configurations is reported as 88.41%, while the 1D CNN model achieved an average accuracy of 85.32%. These results highlight the comparable performance of the LSTM model in accurately identifying road surface conditions when utilizing stacked CA and CD coefficients.

**Table 6:** Comparing five-folds cross-validation average results for LSTM and CNN models using DWT.

Class	CA and CD concatenated						CA and CD stacked					
	LSTM			1D CNN			LSTM			1D CNN		
	Avg. precision	Avg. recall	Avg. F1-score	Avg. precision	Avg. recall	Avg. F1-score	Avg. precision	Avg. recall	Avg. F1-score	Avg. precision	Avg. recall	Avg. F1-score
Normal road	82.8	94.23	88.11	88.14	92.54	90.22	90.36	92.79	91.53	89.53	90.38	89.88
Potholes	85.94	92.67	89.02	94.3	96	94.98	93.85	97.29	95.42	96.55	90.63	93.3
Speedbumps	73.45	78.26	75.57	86.59	81.7	83.93	83.82	90.04	86.74	81.24	85.58	83.16
Bad road	87.76	64.47	73.96	82.13	79.62	80.76	88.91	79.3	83.72	81.07	77.25	78.87
macro avg	82.49	82.41	81.66	87.79	87.47	87.48	89.24	89.86	89.35	87.1	85.96	86.3
Avg. accuracy	81.43%			86.4%			88.41%			85.32%		
Std	0.0235			0.0145			0.0101			0.0202		

The results presented in Table 7 illustrate the performance of 2D CNN models using CWT with a fivefold cross-validation. The results indicate that the models have good accuracy, with an average of 87.7%, and good performance in identifying road conditions, particularly potholes and normal road surfaces. Moreover, the standard deviation is relatively low, indicating consistent performance.

**Table 7:** Five-folds cross-validation average results for 2D CNN models using CWT frequency domain representation.

Class	Avg.precision	Avg.recall	Avg. F1-score
Normal road	91.13	91.7	91.33
Potholes	92.65	92.65	92.38
Speedbumps	82.76	89.64	86.01
Bad road	86.7	79.77	82.98
macro avg	88.31	88.44	88.17
Avg. accuracy	87.7%		

### Proposed method

The below table (Table 8) presents the five-fold cross-validation average accuracy for the proposed model, which utilizes frequency domain and time-frequency domain data representations. The average accuracy of the model across all classes is reported as 93.4% with a standard deviation of 0.0177, which indicates that the performance of the model was consistent across different folds of the data. Overall, the model demonstrated good performance across all classes, with high precision, recall, and F1-scores for Normal roads and potholes and reasonably good performance for speedbumps and Bad roads.

**Table 8:** Five-folds cross-validation average results for the proposed model.

Class	Avg.precision	Avg.recall	Avg. F1-score
Normal road	95.39	96.03	95.69
Potholes	93.04	96.67	94.76
Speedbumps	92.33	92.69	92.5
Bad road	91.63	89.57	90.53
macro avg	93.1	93.74	93.37
Avg. accuracy	93.4%		
Std	0.0177		

**Figure 13:**
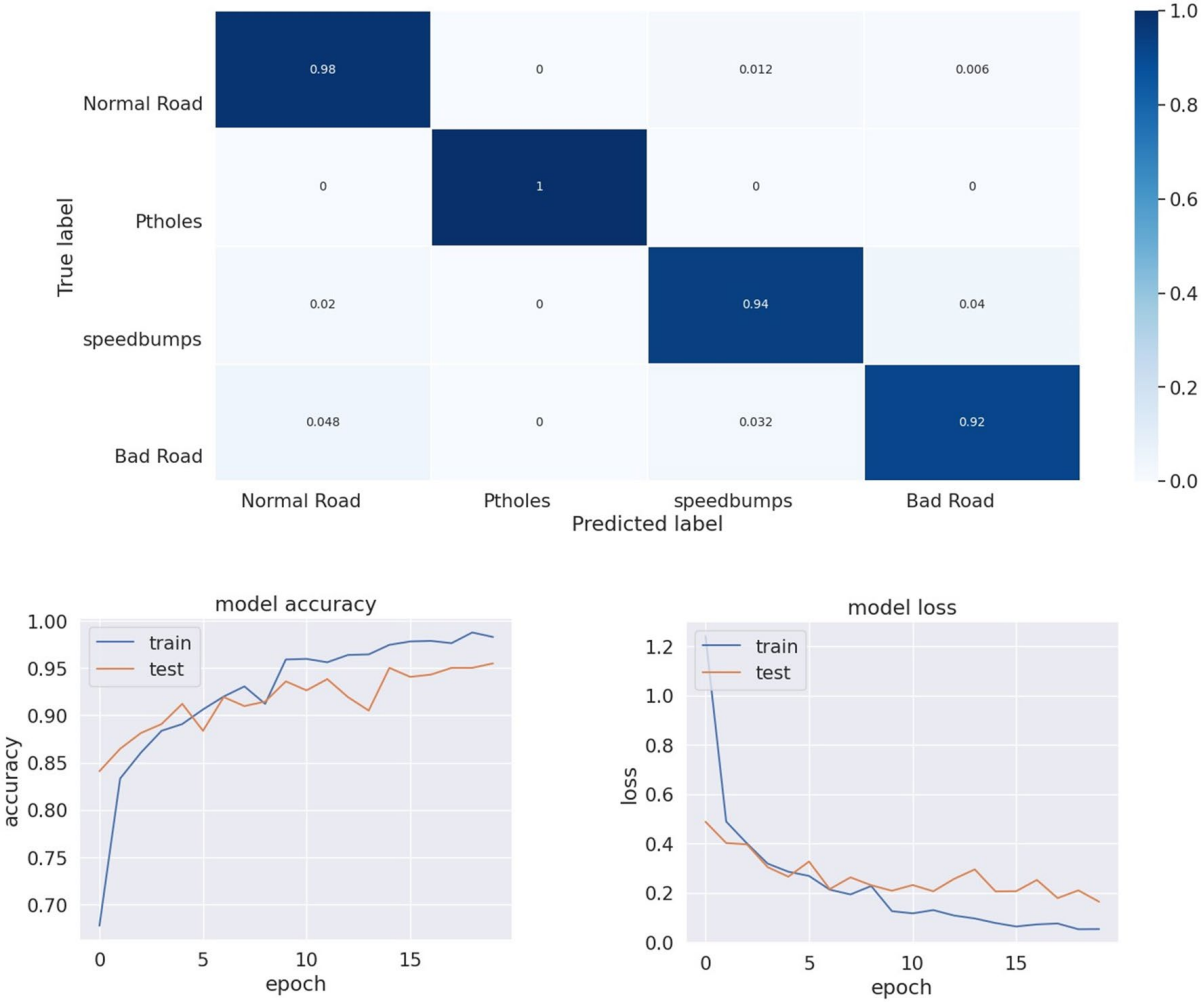
Proposed model performance.

Figure 13 represents the proposed model performance for the optimal fold. The accuracy graph illustrates a steady increase in accuracy as the number of training iterations increases, suggesting that further training could lead to even better results. Also, the loss graph shows a consistent decrease, indicating that the model is effectively learning and minimizing errors. Overall, these performance metrics indicate that the proposed model is capable of achieving high accuracy and robustness in its predictions.

**Figure 14:**
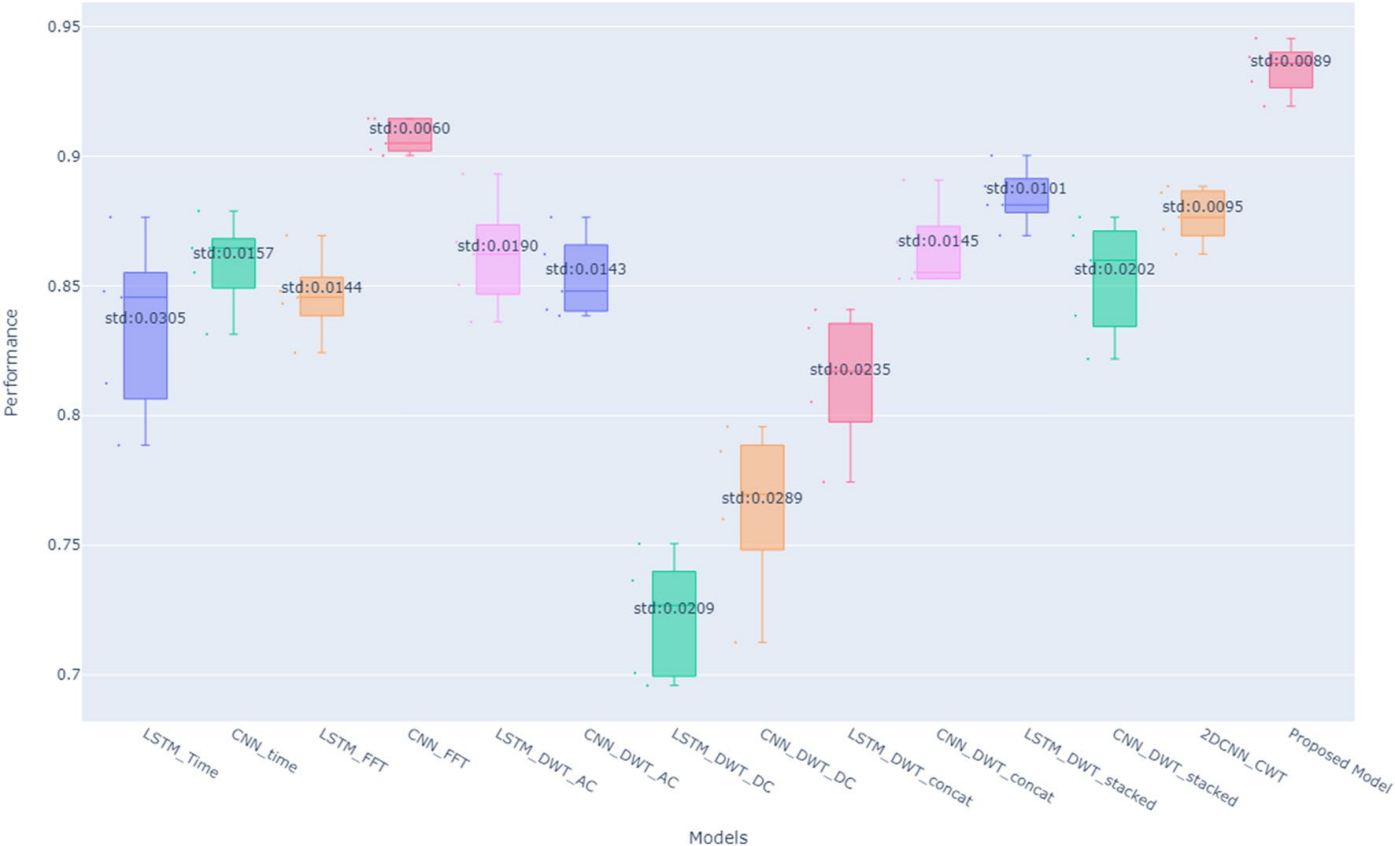
Comparing the average accuracy of the different data representations.

In summary, Based on the results obtained from the comparative analysis of data representation techniques for classifying road surface conditions, the CNN model outperforms the LSTM model in the time and frequency domains. The CNN model is able to extract more meaningful features from the data and has a higher accuracy, F1-score, recall, and precision. Specifically, when using frequency domain representation and 1-D CNN, the model performs the best. On the other hand, LSTM outperforms the CNN model in the time–frequency domain using DWT (AC and DC stacked). The proposed model combined the strengths of CNN using frequency representation and LSTM using time–frequency representation to achieve better results in classifying road surface conditions as shown in Fig. 14.

Table 9 provides a comparison of various models in terms of computational efficiency, represented by the number of floating-point operations (#Flops) and the number of parameters (#Parameter). We used keras_flops library to get the number of Flops and the keras library to calculate the number of parameters. The input shapes for each model are also provided, indicating the dimensions of the input data in different domains and defined as (n_samples, n_timesteps, n_signals). In terms of number of parameters, CNN models are more computationally efficient than LSTM models, making them a better choice for real-time applications. Otherwise LSTM models perform a smaller number of multiplications and additions than CNN.

**Table 9:** Comparison of different models in terms of computational efficiency and input shape.

Domain		Model	#Flops	#Parameter	Input shape
Time		LSTM	1492	69,636	(2105,74,6)
		1D CNN	4,511,768	38,340	
Frequency		LSTM	1276	69,636	(2105, 38, 6)
		1D CNN	1,784,344	34,756	
Time–frequency	DWT (AC or DC)	LSTM	1288	69,636	(2105, 40, 6)
		1D CNN	1,913,752	34,756	
	DWT (CA and CD Concatenated)	LSTM	1528	69,636	(2105, 80, 6)
		1D CNN	4,511,768	38,340	
	DWT(CA and CD Stacked)	LSTM	1528	72,708	(2105, 40, 12)
		1D CNN	2,389,912	42,436	
	CWT	2D CNN	17,880,536	64,132	(2105,74,74,6)
Frequency + time–frequency		Proposed model	1,870,904	150,020	(2105, 38, 6)
					(2105, 40, 12)

Our experiments revealed a clear influence of data representation on classification accuracy. Each domain offers distinct advantages and limitations:

- *Time* *domain*: CNNs performed well, capturing raw signal variations across all road types. However, this domain might be susceptible to noise that can degrade performance.
- *Frequency* *domain*: 1D CNNs utilizing frequency domain data achieved competitive results, suggesting that dominant frequencies hold valuable information for differentiating road surfaces.
- *Time*–*frequency* *domain*: DWT and CWT techniques provided a combined view of both time and frequency characteristics. Notably, LSTMs with stacked DWT approximation and detail coefficients achieved superior performance. This highlights the benefit of capturing both temporal dynamics and spectral information.

Across all domains, CNN models generally outperformed LSTMs in terms of accuracy, F1-score, recall, and precision. This can be attributed to CNNs' ability to effectively learn spatial features from domain representations, making them better suited for identifying patterns in road surface vibrations.

Building on these findings, we propose a novel model that leverages both LSTM and CNN architectures. The model utilizes frequency domain data for the CNN and stacked DWT coefficients for the LSTM. This combination aims to capture the strengths of both time–frequency analysis and dominant frequency extraction. The proposed model achieved a remarkable average accuracy of 93.4% with a low standard deviation, demonstrating its effectiveness.

While CNNs demonstrate superior performance, their computational cost must be considered. As expected, CNN models generally require more resources compared to LSTMs. The selection of the optimal model might depend on the specific application's requirements, balancing accuracy with computational efficiency constraints.

To ensure our findings are reliable and generalizable, we further evaluated the proposed model on another dataset with varying characteristics. In Ref.^29^, the authors collected nine datasets named Passive Vehicular Sensors Dataset (PVS 1–9) using Raspberry Pi and MPU-9250 modules, external GPS, and a camera. They recorded various measurements, including acceleration, gyroscope, magnetometer, temperature, location, and speed data, using two MPU-9250 modules, which were distributed in the vehicle. After preprocessing, the data were used to train and test 34 different computational models for road surface type classification, encompassing both classical machine learning and deep learning techniques. Through extensive experiments, they determined that the best-performing model was CNN-based, achieving a validation accuracy of 93.17%. This model successfully classified road surfaces into categories like dirt, cobblestone, or asphalt roads.

**Figure 15:**
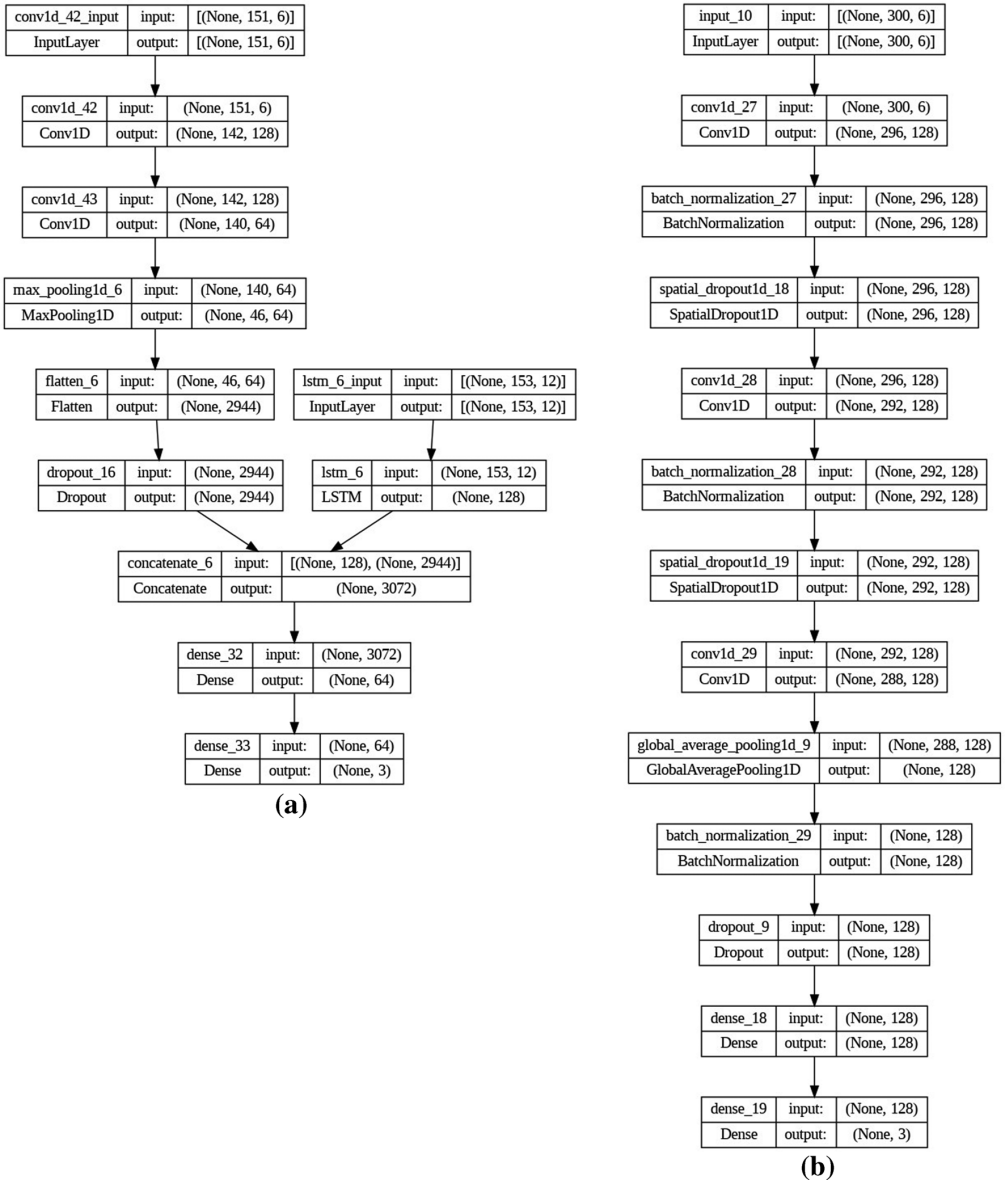
(**a**) Proposed model and (**b**) Ref.^29^ best performance model structure.

We use only The Experiment 3 dataset collected from the module which was attached to the vehicle dashboard and used only the 3-axis acceleration and 3-axis gyroscope. The shape of the input is (4652, 300, 6) for the train dataset and (2546, 300, 6) for the test dataset. To fit the requirements of our proposed model, we apply frequency and time–frequency transformations for the input data, which result in (4652, 153, 12) and (4652, 151, 6), respectively, for training both LSTM and CNN, as shown in Fig. 15.

We compared the performance of our proposed model against their best-performing model to evaluate its effectiveness. The results showed that our model achieved a validation accuracy of 94.8%, outperforming their best-performing model, which indicates the effectiveness of our approach to accurately classifying multivariate time series data.

**Figure 16:**
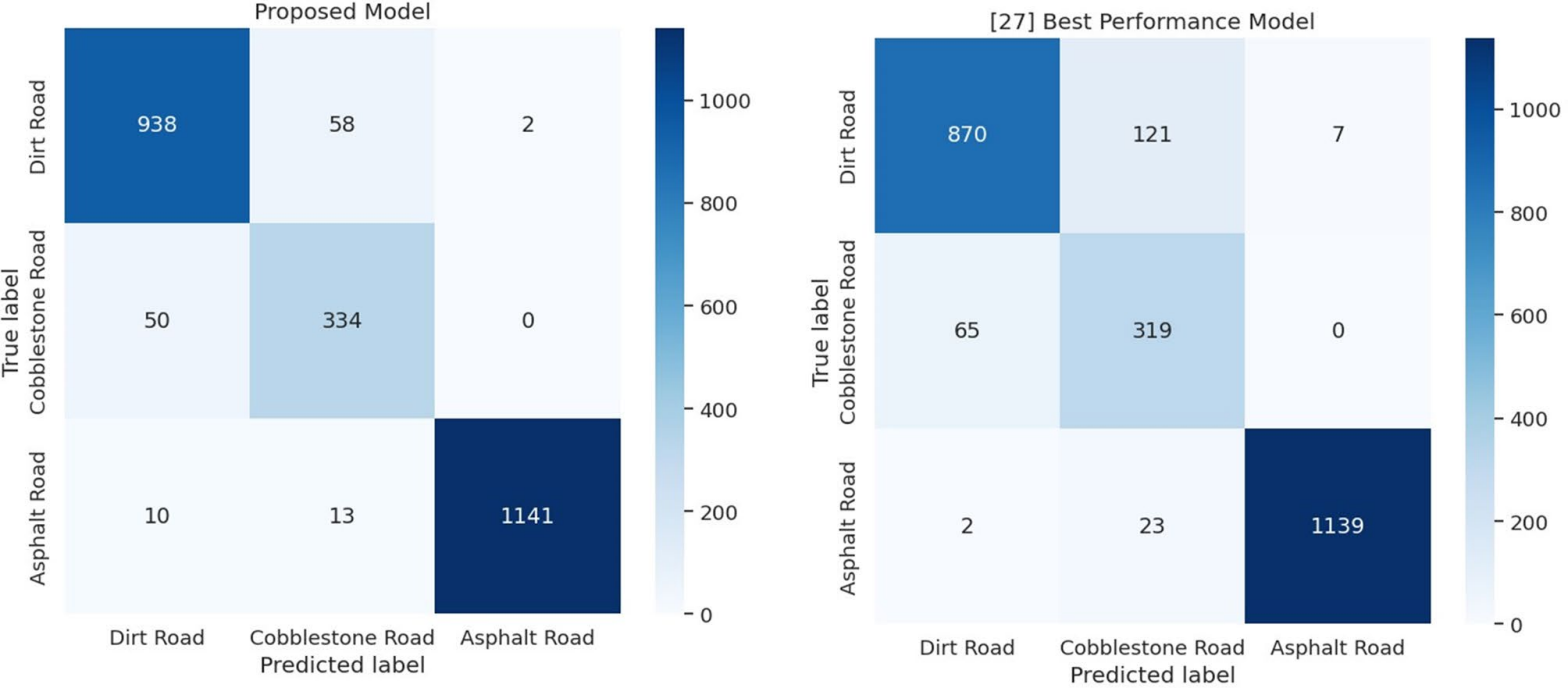
Confusion matrix of the proposed model and Ref.^29^ best performance model.

We compared the performance of our proposed model against^29^ best-performing model to evaluate its effectiveness. The results showed that our model achieved a slightly higher overall accuracy (94.78%), outperforming their best-performing model (91.44%), which indicates the effectiveness of our approach to accurately classifying multivariate time series data as shown in Fig. 16.

Overall, the findings of this study emphasize the significance of selecting appropriate data representation techniques for road surface condition classification. The combination of different representation domains provides a comprehensive understanding of road surface vibrations and enhances classification accuracy.

## Conclusion

In conclusion, this study presents a comparative analysis of data representation techniques for vibration-based road surface condition classification. The findings highlight the strengths and limitations of different representation domains and machine learning models. Furthermore, our research highlights the importance of feature engineering in improving classification accuracy. By extracting relevant features, we were able to enhance the discriminative power of our models and contribute to advancing the field of road surface condition assessment and classification, enabling improved road safety and maintenance strategies.

## Data Availability

The datasets generated during and analyzed during the current study are available from the corresponding author on reasonable request.
